# The impact of aquatic exercise programs on the systemic hematological and inflammatory markers of community dwelling elderly: A randomized controlled trial

**DOI:** 10.3389/fphys.2022.838580

**Published:** 2022-09-28

**Authors:** Carlos Farinha, José Pedro Ferreira, João Serrano, Hélder Santos, Bárbara Oliveiros, Fernanda M. Silva, Márcio Cascante-Rusenhack, Ana Maria Teixeira

**Affiliations:** ^1^ Research Unit for Sport and Physical Activity, University of Coimbra, Coimbra, Portugal; ^2^ Municipality of Sertã, Sertã, Portugal; ^3^ Sport, Health and Exercise Research Unit (SHERU), Polytechnic Institute of Castelo Branco, Castelo-Branco, Portugal; ^4^ Polytechnic of Coimbra, ESTeSC, Clinical Physiology, Coimbra, Portugal; ^5^ Coimbra Institute for Clinical and Biomedical Research, Faculty of Medicine, University of Coimbra, Coimbra, Portugal; ^6^ School of Physical Education and Sports, University of Costa Rica (UCR), San José, Costa Rica

**Keywords:** physical exercise, aquatic environment, hydrogymnastics, ageing, interleukins, inflammageing

## Abstract

Evidence shows that physical exercise is important in maintaining an efficient immune system during ageing. However, there are few studies that test the impact of aquatic exercise programs on the immune system. This study aims to analyze the impact of different physical exercise programs in aquatic environment on the systemic hematological and inflammatory markers of community dwelling elderly. One hundred and two elderly were randomly allocated into four groups: a continuous aerobic exercise group (AerG) (*n* = 25, 71.44 ± 4.84 years); an interval aerobic exercise group (IntG) (*n* = 28, 72.64 ± 5.22 years); a combined exercise group (ComG) (*n* = 29, 71.90 ± 5.67 years); a control group (CG) (*n* = 20, 73.60 ± 5.25 years). The AerG, IntG and ComG participants took part in three different aquatic exercise programs over a 28-weeks period. The CG participants maintained their usual routines during the same time period. Blood samples were collected from all participants in order to access hematologic indicators, by means of cell count, and the inflammatory profile by ELISA. After 28 weeks, significant differences were found for several hematologic variables in the AerG, IntG and ComG with increases in mean corpuscular hemoglobulin (MCH), mean corpuscular hemoglobulin concentration (MCHC), and hemoglobulin (Hb). Decreases in TNF-α levels were found for all exercising groups. An increase in IL-10 levels, granulocytes to lymphocytes ratio (GLR) and a decrease in the TNF-α/IL-10 ratio, were found for the IntG. For the ComG decreases were also found for the TNF-α, IL-1ß/IL-1ra ratios. The present study suggests that aquatic exercise programs were able to improve the inflammatory profile of the participants. Those in the exercise intervention groups showed a shift towards lower pro-inflammatory levels while the non-exercising group showed the opposite behaviour. The IntG and the ComG aquatic exercise programs appeared to be more effective than the AerG program in decreasing chronic low-grade inflammation by mediating the production of higher levels of anti-inflammatory cytokines. However, the differences found between the exercising groups were small and may not have clinical significance.

## 1 Introduction

The immune system is characterized as a complex network of cells and molecules that operate to protect the body from disease, prevent the entry of invading microorganisms and facilitate wound healing (Simpson et al., 2015).

Ageing is associated with immunosenescence, i.e., a progressive decline in the immune function, which causes changes in innate and adaptive immunity. These changes are related to increased morbidity from infectious agents, and the appearance of several age-related diseases, including cardiovascular and metabolic diseases (Sellami et al., 2018). Immunosenescence is characterized by the following aspects: decreased response to new invading infectious agents; unsupported memory T cell response; a higher susceptibility to autoimmune diseases; low-grade chronic inflammation (Rodriguez et al., 2020). A decrease in naive T cells and the consequent increase in memory T cells (Weltevrede et al., 2016) is also well documented. This reduction is considered to be a crucial factor in decreasing the ability to recognize pathogens, which increases the probability of infection (Spielmann et al., 2011). T cells are subject to continuous remodeling, that results from their interaction with different stressors from the internal and external environment. This interaction leads to the decrease of T cells, which is accompanied by a decline in the T cell receptor clonal diversity and consequent increase in the memory cell subpopulation, with an accumulation of terminally differentiated T cells that are dysfunctional or depleted (Rodriguez et al., 2020). Another important aspect of ageing is the development of sterile chronic low-grade inflammation, called inflammaging, which contributes to the pathogenesis of age-related diseases (Franceschi et al., 2018). The TNF-α/IL-10 and IL1ß/IL-1ra ratios are markers that depict the balance between important pro- and anti-inflammatory cytokines (Nisansala et al., 2021) and are associated with the development of coronary and inflammatory diseases (Kumari et al., 2018). More recently the neutrophil to lymphocyte ratio (NLR), the platelet to lymphocyte ratio (PLR) and the systemic immune-inflammation index (SII = NLR x platelets) are being used in the clinical context and provide a multifactorial view of inflammatory processes, with their implementation being increasingly common as inflammatory and prognostic markers of various pathologies such as neurological and cardiovascular diseases. (Walzik et al., 2021).

Regular physical exercise is associated with a reduction of different types of chronic diseases associated with ageing, by means of changes in immune function, specifically in the decrease of immune senescence, the increasing of the innate immune function and the decreasing of chronic inflammation (Abd El-Kader and Al-Shreef, 2018). Continuous exercise of moderate intensity and short duration is associated with an improvement in immune defense. On the other hand, high intensity exercise can cause transient negative changes in immune cell count and function, which can trigger an increased risk of infectious diseases (Souza et al., 2021). Interval exercise programs have been gaining popularity. According to the study by Souza et al. (2021), an isolated session of interval exercise can cause a transient disturbance in the immune system leading to a reduction in immune function. However, the regular practice of programs of interval exercise can induce beneficial adaptations in short- and long-term immune function without altering immune cell counts.

In contrast, a sedentary lifestyle leads to accumulation of visceral fat, which is infiltrated by pro-inflammatory macrophages and T cells. This causes pro-inflammatory macrophages to predominate and the inflamed adipose tissue to release pro-inflammatory cytokines like TNF-α, IL-1β, chemokines and increased expression of Toll like receptors (TLR), which leads to a state of meta-inflammation causing insulin resistance and metabolic disease (Kruger, 2017). In comparison, because physical exercise reduces visceral fat, it will stimulate a decrease in the production and release of adipokines, causing an anti-inflammatory environment during each exercise session (Gleeson et al., 2013). In addition to the reduction of visceral fat, physical exercise, more specifically skeletal muscle contractions, directly stimulates the production of IL6, that will produce anti-inflammatory cytokines, such as IL-10, IL-1ra, and cortisol secretion (Pedersen & Fischer, 2007).

A study by Chupel et al. (2017) concluded that a 28-weeks intervention with a muscular strength exercise program supported the increase of anti-inflammatory balance in a sample of older women. Similar results were observed by Furtado et al. (2020), after an intervention with two different physical exercise programs (a combined exercise program, with muscle strength exercise using elastic bands and a chair-based combined exercise). In another study by Werner et al. (2019), the results showed an increase in leukocytes, granulocytes and lymphocytes after a 26-weeks intervention with aerobic exercise and interval aerobic exercise. Contrastingly, the muscle strength exercise did not produce the same increase. However, Kapasi et al. (2003) concluded that an 8-month intervention with combined exercise (aerobic exercise and muscle strength exercise) did not induce changes in lymphocyte subpopulations. Likewise, Shimizu´s et al. (2011) study showed that the number of leukocytes, lymphocytes and monocytes did not change after a 12-weeks muscle strength intervention program.

The studies mentioned above have shown that physical exercise can be a powerful tool to improve the inflammatory profile of elderly participants. However, it is still not clear which type of exercise is the most effective. Additionally, few studies have analyzed the impact of physical exercise programs within these indicators, in an aquatic environment (Bansi et al., 2013; Santos, 2020). Considering the limitations previously mentioned, the purpose of this study is to compare the impact of different physical exercise aquatic programs (continuous aerobic exercise, interval aerobic exercise and combined exercise) on the systemic hematological and inflammatory markers of community dwelling elderly, and determine which of the three aquatic exercise programs used was the most effective.

## 2 Materials and methods

### 2.1 Study design

A randomized controlled trial was conducted in the Beira Interior region, Portugal. A sample of non-institutionalized elderly participants were submitted to 28-weeks aquatic exercise intervention (October-May). The entire study protocol was previously published (Ferreira et al., 2020). To analyzed the impact of different aquatic exercise programs on the immunologic profile of elderly participants, three different physical exercise programs were used: continuous aerobic (AerG), interval aerobic exercise (IntG) and combined exercise (ComG). Blood samples were collected at two specific time moments, namely: pre-intervention (baseline, M1) and post-intervention (after 28 weeks, M2). This study was carried out according to the recommendations of the Declaration of Helsinki for Human Studies. The protocol was approved by the Ethics Committee for Health of the Faculty of Sport Sciences and Physical Education, University of Coimbra (reference: CE/FCDEF-UC/00462019). Written informed consent was obtained from all participants prior to any protocol-specific procedures.

### 2.2 Participants and sample size

The size and statistical power of the sample were calculated using the G*Power software application (Franz et al., 2007). The following parameters were considered: F test (ANOVA); effect size: 0.25; α-level: 0.05; statistical power: 0.95; number of groups: 4; number of measures: 2 (pre and post intervention); margin of possible losses and refusals: 30%. Therefore, the initial size of the total sample was estimated at 76 participants.

Initially, 174 individuals from the community were invited to participate in the study. After the application of the inclusion and exclusion criteria, 152 individuals were randomized into four groups: continuous aerobic exercise group (AerG, n = 36); interval aerobic exercise group (IntG, n = 41); combined exercise group (ComG, n = 48); control group (CG, n = 27). According to the experience of the research team and previous studies, the dropout rate from exercise programs among older populations is high, so we recruited more participants to compensate for potential dropouts (Dziubek et al., 2015; Chupel et al., 2017; Moreira et al., 2020). A simple randomization method was used. An external investigator used a computer-generated list of random numbers to allocate participants to each group. The investigators were blinded for the randomization of the groups.

The following inclusion criteria were applied: 1) participants from both sexes; 2) 65 years old or more; 3) non-institutionalized; 4) autonomy to move from their residence to Sertã municipal swimming pool; 5) filling out the informed consent form; 6) individuals with medical authorization to practice physical exercise in an aquatic environment, in the cases where some type of clinical condition or comorbidity was present. The following exclusion criteria were also defined: 1) individuals with clinically diagnosed pathologies putting their health and others at risk while doing physical exercise in an aquatic environment; 2) individuals that obtained a score of less than 9 points in the Mini-Mental State Examination (indicating severe cognitive impairment) or were clinically diagnosed with a mental illness.

The AerG, IntG and ComG groups performed physical exercise in an aquatic environment during the same period of 28 weeks (each group took part in a different exercise program). Participants from the CG group were asked to maintain their normal daily activities, without performing any type of systematic physical exercise during the intervention period.

**FIGURE 1:**
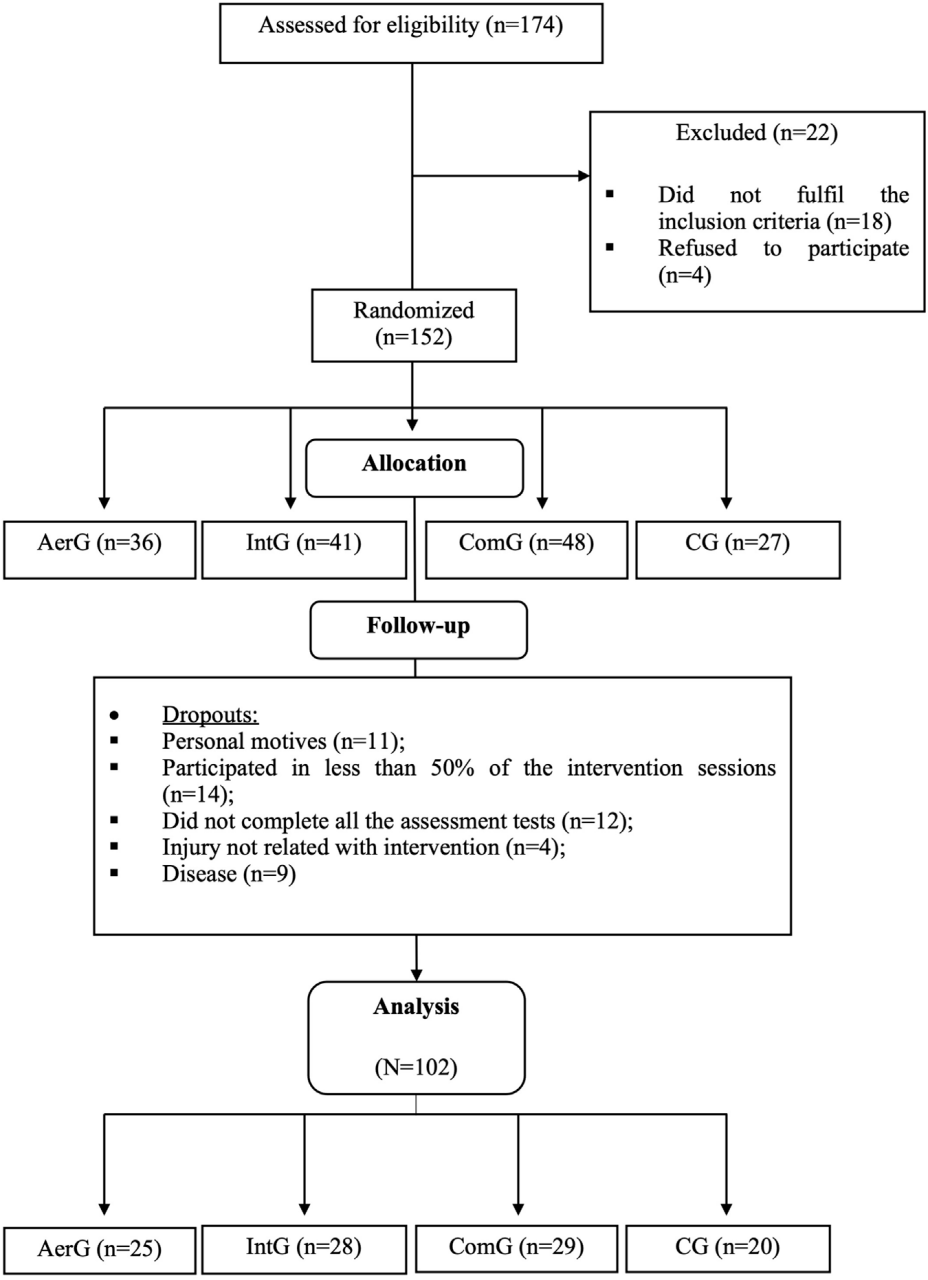
Allocation process for the different groups: Continuous aerobic group (AerG); Aerobic interval group (IntG); Combined group (ComG); Control group (CG).

Fifty participants dropped or were excluded out from the study due to the following reasons: personal reasons (n = 11); less than 50% of attendance of the exercise sessions (n = 14); did not complete all the assessment tests (n = 12); injury not related with the exercise intervention (n = 4); disease (n = 9). Consequently, 102 participants completed the entire process (AerG: n = 25, 71.44 ± 4.84 years, 80% female; IntG: n = 28, 72.64 ± 5.22 years, 89.3% female; ComG: n = 29, 71.90 ± 5.67 years, 75.9% female; CG: n = 20, 73.60 ± 5.25 years, 55% of females). Figure 1 shows the entire allocation process for the different groups.

### 2.3 Outcomes measurements

#### 2.3.1 Sample characterization

Data such as age, regular medication, allergies, alcohol consumption, smoking and medication habits were also obtained in the first phase of data collection (M1) by completing a specific questionnaire. Values referring to anthropometry and functional fitness were also collected. Height (Hgt) was assessed using a portable Seca Bodymeter® stadiometer (model 208, Hamburg, Germany) with an accuracy of 0.1 cm. Weight (Wgt), body mass index (BMI), visceral fat (VF), fat mass (FM) and muscle mass (LBM) were evaluated using the TANITA BC-601 impedance scale (Tokyo, Japan). Functional fitness was assessed using the following tests from the Senior Fitness Test set, developed by Rikli and Jones (1999) and validated for the Portuguese population by Baptista and Sardinha (2005): muscle strength of the lower (MI) and upper (MS) limbs, with the Chair Stand test (30s-CS) and Arm Curl test (30s-AC), respectively (repetitions/30s); aerobic capacity, with the Two-Minute Step test (2min-ST) (nº. of steps); the flexibility of MI and MS, with the Chair Sit and Reach test (CSR) and Back Scratch test (BS), respectively (centimeters); agility and dynamic balance, with the Timed Up and Go test (TUG) (seconds). The handgrip strength was also evaluated, with the Hang Grip test (HG), using the Jamar hand dynamometer (Lafayette Instrument Company, United States) (kg). These data are presented in Table 1.

**TABLE 1:** Participant’s baseline characteristics by group.

	AerG (*n* = 25)	IntG (*n* = 28)	ComG (*n* = 29)	CG (*n* = 20)	*p*-value
	Mean (SD)	Mean (SD)	Mean (SD)	Mean (SD)	Mean (SD)
Morphological parameters					
Age (years)	71.44 (4.8)	72.64 (5.2)	71.90 (5.7)	73.60 (5.3)	0.504
Stature (m)	1.58 (0.7)	1.56 (0.7)	1.57 (0.7)	1.60 (0.9)	0.331
Body mass (kg)	70.5 (8.1)	71.3 (14.3)	75.1 (11.0)	75.5 (13.3)	0.334
BMI (kg/m^2^)	28.20 (3.3)	29.10 (4.8)	30.80 (5.3)	29.50 (5.8)	0.272
VF (%)	11.0 (3.0)	12.0 (3.0)	13.0 (3.0)	13.0 (6.0)	0.128
FM (%)	38.9 (7.3)	41.0 (6,7)	40.3 (9.8)	34.9 (10.9)	0.134
LBM (%)	26.5 (4.3)	24.5 (3.0)	25.5 (4.3)	27.7 (4.7)	0.079
Physical fitness tests					
2min-ST (no of steps)	80.9 (17.4)	71.5 (16.5)	81.6 (19.2)	74.3 (18.9)	0.069
CSR-R (cm)	-0.5 (6.6)	-3.7 (10.6)	-3.5 (7.8)	-7.6 (9.7)	0.099
CSR-L (cm)	0.6 (7.2)	-3.9 (9.9)	-5.8 (9.9)	-3.5 (7.3)	0.054
BS-R (cm)	-9.9 (10.4)	-11.9 (11.5)	-14.3 (9.7)	-16.6 (9.9)	0.157
BS-L (cm)	-14.4 (7.2)	-17.4 (8.6)	-21.0 (10.8)	-20.6 (10.7)	0.056
TUG (s)	6.1 (1.1)	7.4 (1.8)	7.4 (3.0)	6.8 (1.7)	0.110
30s-CS (reps/30s)	15.0 (3.0)	13.0 (4.0)	13.0 (3.0)	15.0 (5.0)	0.185
30s-AC (reps/30s)	21.0 (6.0)	17.0 (7.0)	20.0 (5.0)	19.0 (6.0)	0.119
HG-R (kg)	22.0 (6.0)	21.0 (9.0)	21.0 (9.0)	24.0 (9.0)	0.411
HG-L (kg)	21.0 (6.0)	20.0 (9.0)	21.0 (9.0)	21.0 (10.0)	0.578
Health parameters	N (%)	N (%)	N (%)	N (%)	
Regular medication	23 (92%)	27 (96%)	26 (90%)	19 (95%)	0.768
No alcohol and smoking habits	22 (88%)	25 (89%)	26 (90%)	19 (95%)	0.772
Allergies	10 (40%)	9 (32%)	14 (48%)	7 (35%)	0.638

SD, standard deviation; AerG, continuous aerobic group; IntG, aerobic interval group; ComG, combined group; CG, control group; Hgt, height; Wgt, weight; BMI, body mass index; VF, visceral fat; FM, fat mass; LBM, muscle mass; 2min-ST, two-minute step test; CSR-R, chair sit and reach test - right; CSR-L, chair sit and reach test - left; BS-R, back scratch test-right; BS-L, back scratch test-left; TUG, timed up and go test; 30s-CS, chair stand test; HG-R, hand grip test-right; HG-L, hand grip test-left. Regular medications include: medication to control blood pressure, cholesterol and diabetes.

#### 2.3.2 Biochemical markers (systemic hematological and inflammatory markers)

Participants were instructed to avoid exercise practice 72 h before blood collection. Fasting Blood samples (15 ml) were collected from the ante cubital vein by a registered nurse. A complete blood count (CBC) was conducted using an automatic hematology analyzer (Coulter Act, Beckman Coulter, United States) to obtain the values for: leukocyte (LEU); lymphocytes (LI); monocytes (MO); granulocytes (GR); erythrocytes (ERI); hemoglobulin concentration (Hb); hematocrit (Hct); mean corpuscular volume (MCV); mean globular hemoglobulin (MCH); mean corpuscular hemoglobulin concentration (MCHC); erythrocyte distribution width (RDW); platelets (PL); mean platelet volume (MPV). Next, the test tubes were centrifuged for 20 min at 800 g and stored in cryogenic test tubes at -80 °C until further use. Levels of interleukin 1 receptor antagonist (IL-1ra), interleukin 1 beta (IL-1ß), interleukin 10 (IL-10) and tumor necrosis factor (TNF-α), were subsequently analyzed by ELISA (Invitrogen®, Alfagene, Portugal) according to the manufacturer instructions.

### 2.4 Intervention protocol

The exercise programs were implemented by Sport Sciences and fitness experts, with specific training in water aerobics and developed according to the exercise prescription guidelines recommended by the American College of Sport Medicine (ACSM) for the elderly (ACSM, 2018).

All exercise programs sessions had a duration of 45 min, twice a week, for 28 weeks and were performed in water environment (the water level was between 0.80 and 1.20 m with a temperature of approximately 32 °C), using the music rhythm as a tool to control the intensity of the exercise. Sequences of aquatic exercises, previously defined and selected according to the objectives of each program, were applied. Water exercise sessions were organized into three different parts: initial, main and final part.

The initial part or warm-up last between 10 and 15 min, at low intensity (30–40% max HR), and was the same in the three water exercise programs. This initial part, was aimed at the participants adaptation to the aquatic environment, i.e., to the water temperature, and provided muscular and metabolic stimulations to prepare the body for the main part of the session. Thus, simple exercises in water were used, such as displacements and isolated movements, with a progressive increase in complexity and intensity throughout the initial part.

**FIGURE 2:**
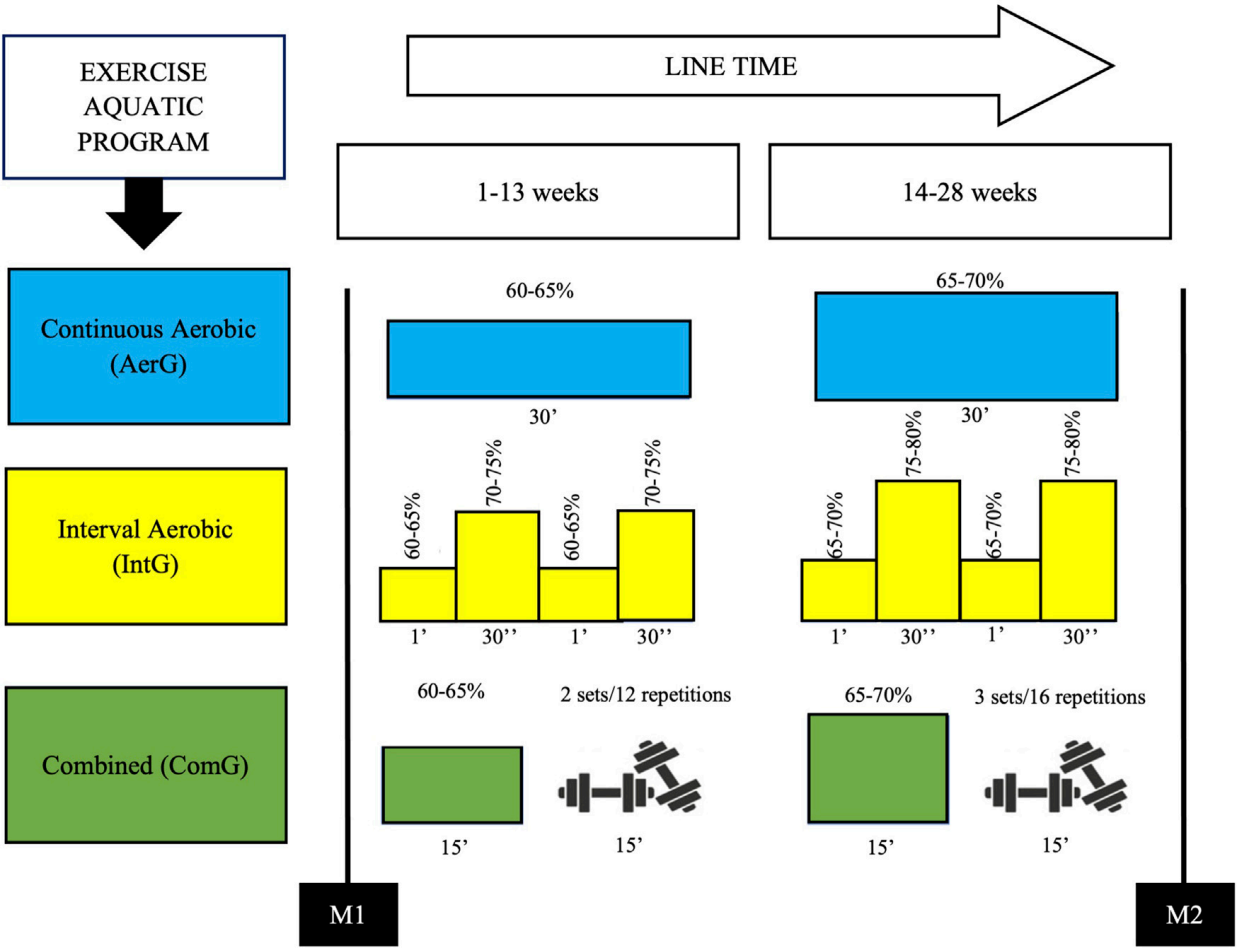
Time line, periodization and characterization of the exercise programs.

The main part of the three water exercise programs sessions had a duration of 20–30 min and were characterized as follows (Figure 2):

- In the water continuous aerobic program (AerG), aerobic exercises were used continuously throughout the main part of the session (20–30 min), with a target intensity of 60–70% of the maximum heart rate, according to the recommendations of the ACSM for the elderly (ACSM, 2018);- In the water interval aerobic program (IntG), the main part of the session consisted of performing exercises with different intensities, such as: short duration exercises of 30 s, with an intensity of 70–80% of the maximal heart rate, followed by active recovery intervals of 1 min, using exercises with an intensity of 60–70% of the maximal heart rate;- In the water combined training program (ComG), the main part of the session was divided into two phases, with equal time periods. The first phase consisted of aerobic exercises on a continuous basis, with a target intensity between 60 and 70% of the maximal heart rate. In the second phase, muscle strengthening exercises were applied (water environment): 6 to 8 different exercises, with auxiliary equipment to create more resistance to movement (e.g., “spaghetti” and dumbbells), covering muscle strengthening of trunk (e.g., rowing, inverted crunch, etc.), upper limbs (e.g., elbow flexion and extension, shoulder rotation, etc.) and lower limbs (e.g., knee flexion and extension, leg abduction and adduction, etc.). Strengthening exercises were implemented using 2 to 3 sets of 12–16 repetitions at moderate intensity (6-7 points in the Borg Scale).

The final part of the water exercise sessions lasted between 5 and 10 min and were similar for all the three water exercise programs. This part consisted of two phases: return to calm, where relaxation exercises were applied to bring back the participants’ heart rate to values close to the resting state, and stretching exercises stimulating a greater range of motion, used to stretch the main muscle groups used throughout the sessions.

### 2.5 Monitoring the exercise programs intensity

For safety reasons and intensity target control, participants randomly used heart rate monitors (Polar, R800CX) during the three water exercise programs sessions (10 heart rate monitors were used per session). Depending on the heart rate values obtained, intensity adjustments to the training plan were performed to maintain the intensity target defined for each water exercise program.

For safety and intensity target control, the intensity of the different water exercise programs was predicted indirectly using the Karvonen´s formula (Karvonen et al., 1957):

Target heart rate=((maximal heart rate−resting heart rate) X % intensity)+resting heart rate

Additionally, and to calculate the maximal heart rate, the Franklin et al. (2000) formula for the elderly was used:

Maximal HR=207 beats per min⁡.−(0.7 X chronological age)

### 2.6 Statistical analysis

The collected data was subjected to descriptive statistical analysis where values such as maximum, minimum, mean and standard deviation were calculated for each variable in each assessment moment. Afterwards, data normality was tested by considering the response to three conditions: z-values from the Skewness and Kurtosis tests; *p*-values from the Shapiro-Wilk test; and visual inspection of generated histograms. All longitudinal comparisons were performed using complete case analysis. Parametric data was analyzed using the Student’s t-test for paired samples to compare the different moments (M1 and M2) and the one-factor Anova test and post-hoc Tukey’s test to analyze the differences between groups both at M1 and M2. Nonparametric data was analyzed using the Wilcoxon test to compare the different moments (M1 and M2) and the Kruskal Wallis and Bonferroni tests to analyze differences between groups. Statistical analysis was performed using the Statistical Package for the Social Sciences (SPSS) statistical software, version 27.0. The level of significance used was *p* ≤ 0.05.

## 3 Results

The results from 102 non-institutionalized elderly participants at baseline, distributed into four different groups (AerG: n = 25, 71.44 ± 4.84 years; IntG: n = 28, 72.64 ± 5.22 years; ComG: n = 29, 71.90 ± 5.67 years; CG: n = 20, 73.60 ± 5.25 years) that completed a 28-weeks intervention are shown in Table 1.

Participants from all groups (AerG, IntG, ComG and CG) showed similar characteristics at baseline, with no significant statistical differences in anthropometrics, physical fitness or clinical characterization variables regarding each group.

The variation in the results for the systemic hematologic markers when analyzed by type of aquatic exercise program, before and after the 28-weeks intervention, are shown in Table 2.

**TABLE 2:** Results of systemic hematological variables at baseline and after 28 weeks of intervention.

	AerG			IntG			ComG			CG				
	M1	M2	Time (p)	M1	M2	Time (p)	M1	M2	Time (p)	M1	M2	Time (p)	Group (M1)	Group (M2)
	Mean (SD)	Mean (SD)		Mean (SD)	Mean (SD)		Mean (SD)	Mean (SD)		Mean (SD)	Mean (SD)			
LEU (×10^3^/μL)	5.99 (1.17)	6.04 (1.26)	0.838	5.85 (1.22)	6.34 (1.26)	0.006*	6.37 (1.37)	6.65 (1.51)	0.044**	5.70 (1.30)	5.95 (1.44)	0.183	0.269	0.335
LI (%)	35.67 (7.57)	36.28 (8.38)	0.657	34.09 (7.54)	32.76 (9.06)	0.158	35.57 (8.19)	34.00 (8.75)	0.140	37.00 (8.23)	35.17 (7.95)	0.108	0.546	0.444
MO (%)	5.76 (2.16)	5.88 (1.80)	0.844	6.39 (2.38)	6.29 (2.06)	0.809	6.57 (1.95)	6.21 (2.25)	0.542	5.99 (2.01)	5.74 (1.79)	0.665	0.505	0.741
GR (%)	58.58 (8.11)	57.84 (8.44)	0.575	59.51 (8.45)	61.23 (9.50)	0.094	57.86 (9.12)	59.79 (8.81)	0.123	57.01 (8.97)	59.23 (9.42)	0.098	0.604	0.396
LI# (×10^3^/μL)	2.13 (0.63)	2.17 (0.55)	0.707	2.00 (0.59)	2.07 (0.66)	0.213	2.26 (0.65)	2.22 (0.66)	0.554	2.06 (0.45)	2.04 (0.49)	0.741	0.529	0.676
MO# (×10^3^/μL)	0.34 (0.14)	0.36 (0.14)	0.503	0.37 (0.16)	0.39 (0.17)	0.388	0.43 (0.16)	0.41 (0.16)	0.503	0.33 (0.13)	0.27 (0.12)	0.078	0.152	**0.013**††
GR# (×10^3^/μL)	3.51 (0.86)	3.52 (1.03)	0.920	3.47 (0.87)	3.89 (1.04)	0.006*	3.71 (1.06)	4.02 (1.27)	0.046**	3.32 (1.04)	3.50 (1.16)	0.236	0.822	0.259
ERI (×10^3^/μL)	4.63 (0.30)	4.53 (0.33)	0.032**	4.59 (0.40)	4.46 (0.32)	0.001**	4.64 (0.27)	4.53 (0.26)	0.003*	4.66 (0.42)	4.62 (0.55)	0.706	0.852	0.611
Hb (g/dL)	13.15 (0.80)	13.23 (0.71)	0.591	13.12 (1.07)	13.27 (0.90)	0.153	13.09 (0.77)	13.38 (1.08)	0.038**	13.22 (0.62)	13.45 (1.04)	0.285	0.793	0.852
Hct (%)	41.66 (2.19)	40.42 (2.21)	0.006*	41.65 (3.03)	40.00 (2.54)	0.000*	42.02 (2.50)	40.75 (2.56)	0.001**	42.20 (2.08)	41.91 (2.72)	0.555	0.897	0.073
MCV (fL)	90.22 (4.24)	89.34 (4.07)	0.041*	90.82 (3.81)	89.97 (3.86)	0.003*	90.75 (3.79)	89.92 (3.84)	0.006*	91.00 (5.51)	90.53 (5.03)	0.068	0.932	0.822
MCH (pg)	28.46 (1.44)	29.24 (1.60)	0.001*	28.60 (1.33)	29.86 (1.47)	0.000*	28.29 (1.43)	29.53 (1.72)	0.000*	28.54 (1.77)	29.19 (2.10)	0.092	0.868	0.477
MCHC (g/dL)	31.54 (0.84)	32.73 (0.77)	0.000**	31.50 (0.71)	33.20 (0.73)	0.000**	31.15 (0.64)	32.83 (1.13)	0.000**	31.37 (0.51)	32.09 (1.45)	0.079	0.155	**0.044**††
RDW (%)	12.86 (0.53)	12.73 (0.49)	0.109	12.88 (0.56)	12.71 (0.72)	0.158	13.23 (0.81)	12.89 (0.78)	0.005*	12.85 (0.50)	12.69 (0.58)	0.202	0.078	0.681
PL (×10^3^/μL)	197.44 (53.91)	198.64 (63.90)	0.467	191.75 (47.50)	187.64 (46.18)	0.190	212.00 (39.28)	206.34 (42.46)	0.347	182.10 (38.28)	173.55 (38.57)	0.079	0.134	0.056
MPV (fL)	8.72 (1.03)	8.46 (0.83)	0.017*	8.70 (0.91)	8.48 (0.84)	0.002*	9.02 (0.91)	8.76 (0.75)	0.000**	8.42 (0.81)	8.28 (0.75)	0.058	0.149	0.154
PLR ratio	98.91 (32.19)	95.05 (30.01)	0.677	102.93 (35.01)	96.33 (27.49)	0.092	102.25 (35.63)	101.18 (35.09)	0.721	93.66 (30.84)	90.39 (28.26)	0.332	0.875	0.749
GLR ratio	1.77 (0.62)	1.71 (0.61)	0.530	1.88 (0.70)	2.06 (0.82)	0.046**	1.78 (0.74)	1.95 (0.78)	0.103	1.67 (0.61)	1.78 (0.64)	0.247	0.610	0.365
SII	351.02 (147.93)	347.65 (187.99)	0.696	350.13 (117.95)	373.89 (129.63)	0.265	383.44 (185.45)	409.24 (182.16)	0.430	309.53 (142.73)	311.83 (138.38)	0.823	0.600	0.178

*Result obtained through T-Student test.

**Result obtained through Wilcoxon test.

†Results obtained through ANOVA e Tukey test.

††Results obtained through Kruskal Wallis e Bonferroni test.

Bold are intended to highlight the variables where statistically significant differences were found.

SD, standard deviation; AerG, continuous aerobic group; IntG, aerobic interval group; ComG, combined group; CG, control group; LEU, Leukocytes; LI, lymphocytes percentage; MO, monocytes percentage; GR, granulocytes percentage; LI#, lymphocytes gross value; MO#, monocytes gross value; GR#, granulocytes gross value; ERI, erythrocytes; Hb, hemoglobulin concentration; Hct, hematocrit; MCV, mean corpuscular volume; MCH, mean globular hemoglobulin; MCHC, mean corpuscular hemoglobulin concentration; RDW, erythrocytes distribution width; PL, platelets; MPV, mean platelet volume; PLR ratio, platelets to lymphocytes ratio; GLR, granulocytes to lymphocytes ratio; SII, systemic immune-inflammation index.

No differences were found for the systemic hematologic variables between groups, before the intervention (M1). Significant differences between groups, after the intervention (M2) were found for MO# (*p =* 0.013) between the CG and the IntG participants (*p =* 0.007), as well as between the CG and the ComG (*p =* 0.002), and between the CG and the AerG (*p* = 0.033). Differences in MCHC (*p =* 0.044) between the CG and the IntG participants (*p =* 0.007) were also detected. In all identified cases, the results were lower for the CG.

**FIGURE 3:**
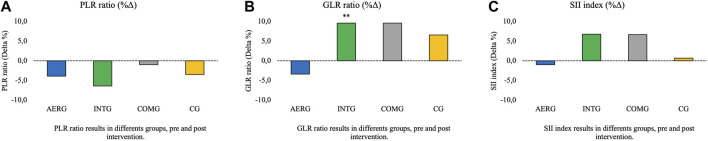
Percentage of delta (%Δ) the difference post-pre intervention for the immune inflammation markers PLR ratio, GLR ratio and SII index.

As a result of the intervention, statistically significant increases were found in the following variables: LEU in the IntG (*p* = 0.006; Δ = 8.4%) and the ComG (*p* = 0.044; Δ = 4.4%); GR# in the IntG (*p* = 0.006; Δ = 12.1%) and the ComG (*p* = 0.046; Δ = 8.4%); MCH in the AerG (*p* = 0.001; Δ = 2.7%), the IntG (*p* < 0.001; Δ = 4.4%) and the ComG (*p* < 0.001; Δ = 4.4%); MCHC in the AerG (*p* < 0.001; Δ = 3.8%), the IntG (*p* < 0.001; Δ = 5.4%) and the ComG (*p* < 0.001; Δ = 5.4%); Hb in the ComG (*p* = 0.038; Δ = 2.2%); and GLR ratio in the IntG (*p <* 0.046; Δ = 9.6%). (Figure 3).

Statistically significant reductions were also found in the following variables: ERI in the AerG (*p* = 0.032; Δ = -2.2%), the IntG (*p* = 0.001; Δ = -2.8%) and the ComG (*p* = 0.003; Δ = -2.4%); Hct in the AerG (*p* = 0.006; Δ = -3.0%), the IntG (*p* < 0.001; Δ = -4.0%) and the ComG (*p* = 0.001; Δ = -3.0%); MCV in the AerG (*p* = 0.041; Δ = -1.0%), the IntG (*p* = 0.003; Δ = -0.9%) and the ComG (*p* = 0.006; Δ = -0.9%); MPV in the AerG (*p* = 0.017; Δ = -3.0%), the IntG (*p* = 0.002; Δ = -2.5%) and the ComG (*p* < 0.001; Δ = -2.9%); RDW in the ComG (*p* = 0.005; Δ = -2.6%). The results for the inflammatory markers analyzed by each type of aquatic exercise program, before and after the intervention, are shown in Table 3.

**TABLE 3:** Inflammatory markers levels at baseline and after 28 weeks of intervention.

	AerG			IntG			ComG			CG				
	M1	M2	Time (p)	M1	M2	Time (p)	M1	M2	Time (p)	M1	M2	Time (p)	Group (M1)	Group (M2)
	Mean (SD)	Mean (SD)		Mean (SD)	Mean (SD)		Mean (SD)	Mean (SD)		Mean (SD)	Mean (SD)			
IL-10 (pg/ml)	18.45 (14.08)	17.67 (15.30)	0.514	28.55 (10.13)	36.70 (6.85)	0.000**	28.15 (14.12)	24.69 (10.04)	0.127	20.32 (12.87)	16.87 (9.10)	0.054	**0.005**††	**0.000**††
IL-1ra (pg/ml)	76.93 (51.79)	81.28 (58.52)	0.757	89.30 (56.68)	92.98 (69.54)	0.585	104.69 (63.82)	115.71 (67.19)	0.157	121.77 (83.56)	88.31 (59.96)	0.008**	0.179	0.220
IL-1ß (pg/ml)	30.05 (3.75)	29.50 (3.31)	0.443	35.04 (16.67)	33.25 (17.15)	0.117	27.98 (10.16)	27.00 (14.27)	0.064	25.39 (4.04)	26.49 (4.24)	0.043*	**0.010**††	**0.007**††
TNF-α (pg/ml)	37.38 (21.07)	28.65 (21.72)	0.011**	17.65 (11.13)	14.27 (9.63)	0.001**	69.01 (28.82)	52.89 (25.25)	0.005*	31.98 (16.75)	32.59 (18.96)	0.936	**0.000**††	**0.000**††
TNF-α/IL-10 ratio	5.00 (6.25)	3.85 (5.66)	0.056	0.89 (1.07)	0.45 (0.52)	0.000**	2.55 (0.75)	2.23 (0.93)	0.125	3.18 (4.16)	3.18 (3.13)	0.351	**0.000**††	**0.000**††
IL-1β/IL-1ra ratio	0.63 (0.45)	0.75 (0.79)	0.657	0.67 (0.60)	1.01 (1.98)	0.982	0.42 (0.40)	0.36 (0.32)	0.047**	0.35 (0.33)	0.50 (0.38)	0.053	**0.025**††	0.097

*Result obtained through T-Student test.

**Result obtained through Wilcoxon test.

†Results obtained through ANOVA e Tukey test.

††Results obtained through Kruskal Wallis and Bonferroni test.

Bold are intended to highlight the variables where statistically significant differences were found.

SD, standard deviation; AerG, continuous aerobic group; IntG, aerobic interval group; ComG, combined group; CG, control group; IL-10, Interleukin 10; IL-1ra, interleukin 1; IL-1ß, interleukin 1 beta; TNF-α, tumor necrosis factor; TNF-α/IL-10, ratio between tumor necrosis factor e Interleukin 10; IL-1β/IL-1ra, ratio between interleukin 1 beta e interleukin 1.

Before the intervention (baseline) statistically significant differences were found between groups in the following inflammatory markers: IL-10 between the AerG and the ComG (*p* = 0.011), the AerG and the IntG (*p* = 0.002), and the IntG and the CG (*p* = 0.022)); IL-1ß between the CG and the AerG (*p* = 0.021), the CG and the IntG (*p* = 0.007), the ComG and the AerG (*p* = 0.045) and the ComG and the IntG (*p* = 0.015)); TNF-α between the IntG and the CG (*p* = 0.004), the IntG and the AerG (*p* < 0.001), the IntG and the ComG (*p* < 0.001), the CombG and the CG (*p* < 0.001), and the ComG and the AerG (*p* = 0.001)); TNF-α/IL-10 between the IntG and the CG (*p* = 0.001), the IntG and the AerG (*p* < 0.001), and the IntG and the ComG (*p* < 0.001); IL-1ß/IL-1ra between the CG and the IntG (*p* = 0.029), the CG and the AerG (*p* = 0.011), and the ComG and the AerG (*p* = 0.035)).

In the identified cases, the results were higher in the IntG and the ComG for IL-10, lower in the ComG and the CG for IL-1ß, lower in the IntG for TNF-α and the TNF-α/IL-10, and lower in the CG and the ComG for IL-1ß/IL-1ra.

After the intervention statistically significant differences were also found between groups in the following variables: IL-10 between the AerG and the ComG (*p* = 0.017), the AerG and the IntG (*p* < 0.001), the CG and the ComG (*p* = 0.042), the CG and the IntG (*p* < 0.001) and the ComG and the IntG (*p* < 0.001)); IL-1ß between the ComG and the IntG (*p* = 0.001), and the ComG and the AerG (*p* = 0.002)); TNF-α between the IntG and the AerG (*p* = 0.001), the IntG and the CG (*p* < 0.001), the ComG and the IntG (*p* < 0.001), the ComG and the AerG (*p* = 0.001), and the ComG and the CG (*p* = 0.036)); TNF-α/IL-10 between the IntG and the AerG (*p* < 0.001), the IntG and the ComG (*p* < 0.001), and the IntG and CG (*p* < 0.001). In the identified cases, the results were higher in the IntG for IL-10 and IL-1ß/IL-1ra, lower in the ComG for IL-1ß, and lower in the IntG for TNF-α.

**FIGURE 4:**
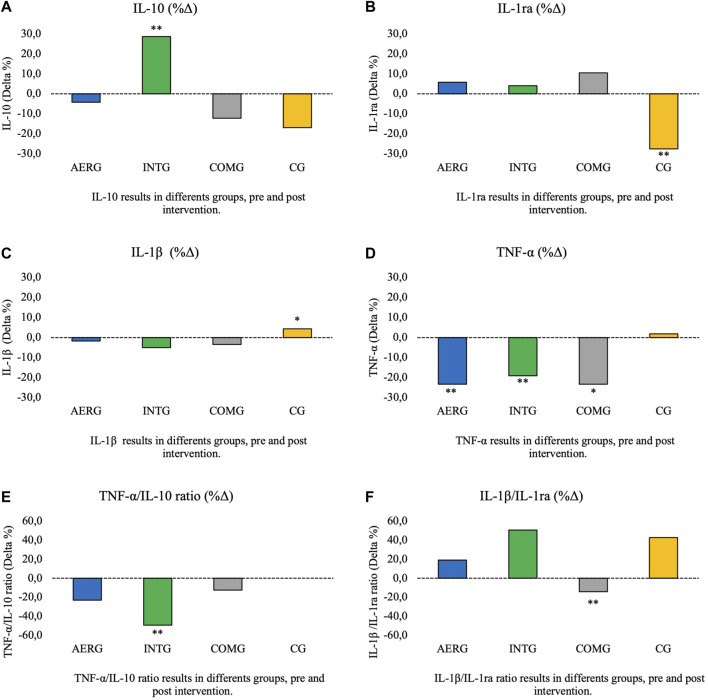
Percentage of difference post-pre intervention for cytokines IL-10, IL-1ra, IL-1β, TNF-α and inflammatory index TNFα-/IL-10, IL-1β/IL-Ira.

The IL-10 levels showed a tendency to decrease in all groups, except for the IntG, which showed a significant statistical increase (*p* < 0.01; Δ = 28.5%). The IL-1ra levels showed a tendency to increase, in all exercise groups, but not in the CG, which showed a significant decrease (*p =* 0.008; Δ = -27.5%). For the IL-1ß levels, a tendency to decrease was found in all aquatic exercise groups, but not in the CG, where there was a significant increase (*p =* 0.043; Δ = 4.3%). The TNF-α levels showed a significant decrease in all exercise groups (AerG: *p =* 0.011; Δ = -23.4%; IntG *p =* 0.001; Δ = -19.1%; and ComG *p =* 0.005; Δ = -23.4%), but not in the CG. The results also showed a significant decrease in TNF-α/IL-10 ratio in the IntG (*p <* 0.001; Δ = -49.3%), and for the IL-1ß/IL-1ra ratio in the ComG (*p =* 0.047; Δ = -13 .5%). (Figure 4).

## 4 Discussion

The purpose of the present study was to evaluate the impact of different aquatic exercise programs (continuous aerobic exercise, interval aerobic exercise and combined exercise) on the systemic hematologic and inflammatory markers of community dwelling elderly. A preliminary systematic bibliographic search revealed the innovative characteristics of the present study, since there were very few studies that had evaluated similar immune variables, in exercise performed in aquatic environment, in older persons.

Results for the systemic hematologic variables revealed significant statistical differences in most of the variables analyzed, due to the intervention with aquatic physical exercise (AerG, IntG and ComG), but no significant differences were found in the CG. A more detailed analysis of the results revealed significant differences over time (from M1 to M2), i.e., between pre- and post-intervention, in the AerG for ERI, Hct, MCV, MCH, MCHC and MPV, in the IntG for LEU, GR, ERI, Hct, MCV, MCH, MCHC and MPV, and in the ComG for LEU, GR, ERI, Hb, Hct, MCV, MCH, MCHC, RDW and MPV. The difference of the mean was higher for MCHC in the AerG (Δ = 3.8%) and for GR in the IntG (Δ = 12.1%) and in the ComG (Δ = 8.4%).

A study carried out by Chupel et al. (2017), that tested the effectiveness of a 28 weeks land-based muscle strength exercise program using elastic bands, in elderly women, showed that exercise intervention caused changes in hematologic variables, specifically significant increases in Hb, MCV, MCH and MCHC, and significant reductions in the LEU and LI variables. In the Hct and ERI variables, no significant statistical differences were found. Similar results had already been reported in Johannsen et al. (2012) study, using a 6-months intervention with aerobic exercise, that promoted significant reductions in the LEU and LI variables. Another recent study by Santos (2020) tested the effectiveness of an aquatic exercise program in elderly women for 1 year, and found a reduction in MCH, MCHC and PL, and an increase in Hct. However, those differences were not statistically significant. The Hb values remained unchanged.

The present study showed similar results to those reported by Chupel et al. (2017) in the three exercise groups (AerG, IntG and ComG), with significant increases in MCH and MCHC. The IntG and ComG groups showed the highest difference in average in both variables. Levels of Hb showed a tendency to increase in the three exercise groups, reaching statistically significance only in the ComG group.

In opposition to the results found in Chupel et al. (2017) study, present results revealed a significant reduction of MCV in the three exercise groups, with the highest variation of the mean being registered in the AerG, and an increase in the number of leucocytes in the IntG and ComG groups. The IntG registered the highest variation of the mean. Regarding the Hct and ERI variables, our results showed a statistically significant reduction in all aquatic exercise groups, with the IntG registering the highest difference of the mean in both cases, in opposition to the results obtained by Chupel et al. (2017). However, in Chupel’s study the participants were much older and frail.

Present results clearly reveal a positive variation regarding hematologic health, with high levels of MCV and ERI being associated with a higher risk of death from all causes, especially cardiovascular and infectious diseases (Dratch et al., 2019). The increase in LEU numbers has positive implications at a hematologic level, since leucocytes have an important role in tissue recovery and host defense as well as antimicrobial properties (D’asta et al., 2018). Despite low levels of Hct being associated with the presence of anaemia, that can lead to the development of several chronic diseases (Suresh et al., 2021), the reduction of Hct values found in our study does not seem to have clinical implications since they remained within the clinical reference values (36–48% for women and 40–54% for men). Our results when compared to those of Santos (2020), found a significant statistical increase in MCH and MCHC, in all exercise groups, and in Hb in the ComG. Thus, present results showed a better effect on hematologic health.

Although some of the present results are somehow different from those found in the literature, the values remained within the normal limits, showing that aquatic exercise contributed to the maintenance of normal hematologic variables. The differences mentioned above may be directly related to the typology of physical exercise programs applied in different studies. In our study, the impact of different aquatic exercise programs (continuous aerobic exercise, interval aerobic exercise and combined exercise) was tested. Our research did not find any other intervention study that included these types of programs or variables, making it an important contribution to this field of research.

Although leucocyte numbers have been shown to increase with ageing (Hopkin et al., 2021), the total amount of LEU in the blood increases with acute physical exercise, i.e., exercise of short duration and high intensity (Gleeson et al., 2013). It is possible that our results may represent a cumulative effect of the acute bouts of exercise performed regularly for 28 weeks.

Regarding the inflammatory markers, significant differences were found for most of the analyzed variables, resulting from the aquatic physical exercise interventions. A more detailed analysis of the results revealed significant differences between the pre- and post-intervention phases for the: AerG, in TNF-α; for the IntG, in IL-10, TNF-α and TNF-α/IL-10; for the ComG, in TNF-α and IL-1β/IL-1ra. The differences of the means for the TNF-α variable was higher in the AerG and the ComG (Δ= -23.4% in both cases).

The results from the Chupel et al. (2017) study showed significant increases in IL-10 after a land-based muscle strength program. In another study published by the same authors (Chupel et al., 2018), a 14-weeks combined exercise program (aerobic exercise and muscle strength exercise) resulted in an increase in the anti-inflammatory markers IL-10 and IL-1ra. For the pro-inflammatory markers, results from the same study revealed a significant reduction in TNF-α and IL-1ß. Another study by Furtado et al. (2020), showed a decrease in IL-1ß after 28-weeks of intervention with a combined exercise program. In aquatic environment, a study carried out by Korb et al. (2018), that tested the effectiveness of a 12-weeks aerobic walking program, showed a reduction in IL-10, while the IL-1ß levels were the same before and after the intervention. In a study by Ortega et al. (2012), that tested the effectiveness of an 8-months aquatic aerobic program, the results showed a decrease for the anti-inflammatory marker IL-10, and for the pro-inflammatory markers IL-1ß and for TNF-α. The results of another study by Da Silva et al. (2018) also showed a reduction in TNF-α after a 12-weeks intervention with a low-intensity aquatic exercise program. Still in an aquatic environment, in the study by Pochmann et al. (2018), the results showed significant increases for IL-1ra after 1 month of intervention with an aquatic physiotherapy program. Colato et al. (2017) applied a 12-week water walking program to a group of overweight women, and the results showed that, after the intervention, both IL-10 and TNF-α values increased significantly. In the same way, statistically significant increases were found for IL-10, after a 12-week aquatic walk/run intervention program (Korb et al., 2018), and TNF-α values also tended to increase after a water bike endurance program intervention (Bansi et al., 2013).

Overall, our results showed a tendency for the reduction in the pro-inflammatory markers IL-1β and TNF-α all the exercising groups. The plasma levels of the anti-inflammatory IL-10 increased in the IntG, while in the other groups the values showed a trend towards a decrease. Similar results were previously found in the studies by Colato et al. (2017) and Korb et al. (2018), after intervention with programs of walking/running in aquatic environment. Athletes do show increased levels of plasma IL-10 in response to high training volume and intensity (Minuzzi et al., 2017). The bouts of high intensity characteristic of the interval aerobic program may explain the differences between the exercising groups for this variable. It is possible that the small decrease of IL-10 found for the AerG and the ComG could also reflect a response to the decrease in TNF-α seen in these groups. As for the IL-1ra levels, our results showed an increasing trend in all exercise groups, whereas in the CG a significant statistical reduction was observed. Similar results had previously been found in the studies by Chupel et al. (2017) and Chupel et al. (2018), after an intervention with a land-based muscular strength and combined exercise program, and in the study by Pochmann et al. (2018), after an aquatic physiotherapy program intervention. Conversely, in the studies by Korb et al. (2018) and Ortega et al. (2012), the IL-10 levels were reduced with the intervention in an aquatic environment (walking exercise and aerobic exercise, respectively). For the IL-1ß variable, our results showed a trend towards a reduction in all exercise groups. However, there was a significant statistical increase in the CG. In the study by Korb et al. (2018), IL-1ß values remained unchanged after a 12-week intervention of walking/running in an aquatic environment. On the other end, for the TNF-α levels there was a significant statistical reduction in all exercise groups (AerG and ComG showed a higher means variation), while in the CG the TNF-α values showed a tendency to increase. Similar results were previously found by Chupel et al. (2018) and Furtado et al. (2020) after land exercise interventions and by Ortega et al. (2012) and Silva et al. (2018) after exercise interventions in aquatic environments. However, the results of Colato et al. (2017) and Bansi et al. (2013) showed increases in TNF-α values after intervention with water walking and water bike endurance, respectively.

The above findings are reflected in the TNF-α/IL-10 inflammatory index tendency to decrease in all aquatic exercise groups, reaching statistically significance in the IntG. For the IL-1β/IL-1ra ratio a statistically significant reduction in the ComG was also found. Our results confirm the results already found in the study by Chupel et al. (2018), where reductions in the TNF-α/IL-10 and IL-1β/IL-1ra ratios also occurred, after intervention with physical exercise on land. No studies were found to assess these ratios in an aquatic environment. Our results suggest that physical exercise in an aquatic environment induces an anti-inflammatory response, reducing chronic low-grade inflammation, and contributing to the reduction in the development of metabolic and cardiovascular diseases. In the study by Kumari et al. (2018), the TNF-α/IL-10 ratio was associated with the risk of coronary artery disease, suggesting that this ratio may play a vital role in the development of this type of pathologies.

Regarding the markers PLR, GLR and SII, few studies were found where the effect of exercise was reported. As for the PLR ratio, our results showed a downward trend in all exercising groups. No intervention studies were found in which the impact of long-term exercise on this marker was evaluated, however, the results found in the studies by Bessa et al. (2016) and Korkmaz et al. (2018), showed that PLR ratio values increased after evaluating the acute effect of physical exercise. Regarding the GLR ratio, our results showed a statistically significant increase for IntG, which is in line with the results found in the study by Svendsen et al. (2016), where high-intensity physical exercise provided significant increases in the NLR ratio. Additionally, our results showed a tendency towards a reduction in the GLR ratio in the AerG, corroborating the results found in the study by Makras et al. (2005), where a moderate-intensity exercise program caused a significant reduction in the NLR values. These data reinforce**s** the idea that higher-intensity physical exercise provides greater increases in NLR ratio, compared to moderate-intensity exercise programs (Walzik et al., 2021). Finally, for SII in the present study there was a visible tendency to increase in the IntG and ComG and to decrease in the AerG. These results contradict those found in the study by Joisten et al. (2021), where SII values were reduced after an intervention with a high-intensity interval exercise program. However, studies that evaluated the acute effect of exercise on SII showed that values increased after higher intensity exercise (Walzik et al., 2021).

Our results showed that physical exercise caused a buffering effect, both in anti-inflammatory and in pro-inflammatory markers, since the results in the exercise groups followed a positive trend towards a less inflammatory environment, while the opposite occurred in the CG where the low-grade chronic inflammation increased. These results provide evidence that aquatic physical exercise programs will improve/mantain the inflammatory profile.

The results of the present study suggest that the three aquatic exercise programs can effectively improve immune variables in community dwelling elderly participants. Some of the changes observed in our study are similar to those found in studies carried out on land environments. Thus, exercise in aquatic environments can be seen as a viable alternative to land-based exercise, especially when other health constraints are installed (e.g. orthopedic, rheumatological or functional limitations, circulatory disease, vertigo, etc.). As for the type of exercise program, the AerG had a higher variation of the mean for TNF-α, the IntG had a higher variation of the mean for IL-10 and IL-1ß, and the ComG had a higher variation of the mean for IL-1ra and TNF-α (the same value as AerG).

## 5 Limitations

Regarding the limitations of the study, a block randomization methodology could have been used, which would have avoided such an unequal number of participants between groups. According to the purpose of the study, the magnitude of the variation should have been tested statistically, giving more robustness to the results. In the statistical procedure we could have applied a repeated-measures ANOVA. However, due to lack of normality and highly skewed data, nonparametric procedure guarantees higher statistical power.

## 6 Conclusion

The results of the present study allow us to conclude that the participation in physical exercise aquatic environment programs can lead to beneficial changes in the systemic hematologic variables of community dwelling elderly.

Regarding the inflammatory markers, the present results showed that, in general, all exercise groups decreased their pro-inflammatory markers levels as well as their inflammatory index TNF-α/IL-10, while the opposite effect was found in the CG. This means that the participation of community dwelling elderly in aquatic physical exercise programs caused a buffering effect, contributing not only to the maintenance, but also to an improvement in their inflammatory profile.

As for the type of aquatic program that best benefited the systemic hematological and inflammatory markers, the results were not totally conclusive, since different exercise programs led to improvements in different variables. All showed important benefits in decreasing chronic low-grade inflammation. However, the combined aquatic exercise program showed significant statistical improvements in a higher number of systemic hematologic variables and a decrease in TNF-α levels, while the interval aerobic aquatic exercise program showed significant statistical improvements in a greater number of inflammatory markers, including an increase in IL-10 and a decrease in TNF-α levels.

Further studies assessing systemic hematologic and inflammatory markers among older participants in physical exercise interventions in aquatic environments are needed, aiming to better clarify the effects of exercise on chronic low-grade inflammation.

## Data Availability

The original contributions presented in the study are included in the article/supplementary material, further inquiries can be directed to the corresponding author.
